# Highly sensitive immunosensing platform for one-step detection of genetically modified crops

**DOI:** 10.1038/s41598-019-52651-2

**Published:** 2019-11-06

**Authors:** Hongfei Gao, Luke Wen, Wei Hua, Jing Tian, Yongjun Lin

**Affiliations:** 1 College of Life Science and Technology, Huazhong Agricultural University, Wuhan, 430070 China; 2 Key Laboratory of Oil Crop Biology of the Ministry of Agriculture and Rural Affairs, Oil Crops Research Institute, Chinese Academy of Agricultural Sciences, Wuhan, 430062 China; 3 MOE Key Laboratory of Environment Correlative Dietology, College of Food Science and Technology, Huazhong Agricultural University, Wuhan, 430070 China

**Keywords:** Analytical biochemistry, Bioanalytical chemistry

## Abstract

The wide cultivation of genetically modified (GM) insect-resistant crops has raised concerns on the risks to the eco-environment resulting from a release of Cry proteins. Therefore, it is vital to develop a method for the quantification of GM crops. Herein, A highly sensitive immunosensing platform has been developed for both colorimetric and chemiluminescent (CL) detection of Cry 1Ab using dual-functionalized gold nanoparticles (AuNPs) as signal amplification nanoprobes for the first time. In this work, anti-Cry 1Ab monoclonal antibody and horseradish peroxidase (HRP) are simultaneously functionalized on the surface of AuNPs with an exceptionally simple synthesis method. Combined with immunomagnetic separation, this immunosensing platform based on colorimetric method could detect Cry 1Ab in one step in a linear range from 1.0 to 40 ng mL^−1^ within 1.5 h, with a limit of detection of 0.50 ng mL^−1^. The sensitivity of fabricated nanoprobes was 15.3 times higher than that using commercial HRP-conjugated antibody. Meanwhile, the fabricated nanoprobes coupled with CL detection was successfully applied for Cry 1Ab detection with a minimum detection concentration of 0.050 ng mL^−1^ within a linear range of 0.10–20 ng mL^−1^. The proposed approach was validated with genuine GM crops, and the results showed a good correlation coefficient of 0.9906 compared to those of a commercial ELISA kit. Compared with ELISA, the developed immunosensing platform significantly simplified the assay procedure and shortened the analytical time, thus providing a new platform for the detection of genetically modified crops with high sensitivity, rapidity and simplicity.

## Introduction

In the past 20 years, genetically modified (GM) crops have been widely cultivated all over the world. Among them, insect-resistant crops are one of the most significant GM crop types, which are developed by introducing *cry* genes isolated from *Bacillus thuringiensis* (*Bt*) into the plant genome. The adoption of *Bt* crops can effectively reduce the usage of pesticides and accelerate the productivity of plants, generating a massive economic benefit. However, the potential risks of *Bt* crops on human health and the eco-environment resulting from the release of Cry proteins remain controversial. To meet the demand for safety control of agricultural GM crops, the labeling of GM products has been mandatory according to a certain labeling threshold in many countries. The implementation of the labeling policy requires the development of a simple, rapid and field-testable analytical approach for *Bt* crop identification and quantification.

Recently, various analytical methods have been developed for GM crop detection, such as the polymerase chain reaction (PCR) assay^1–5^, quartz crystal microbalance biosensors^6^, surface plasma resonance biosensors^7,8^, electrochemical biosensors^9,10^ and electrochemiluminescent biosensors^11^. Although these DNA-based techniques are reliable, accurate and highly sensitive, they require laborious sample pretreatments and expensive instrumentation. An alternative approach for the quantitative detection of GM crops is an antibody-based immunoassay, such as enzyme-linked immunosorbent assay (ELISA)^12^. Despite its low demand for equipment, easy reading and mature application, ELISA requires multiple incubation, separation and washing steps, moreover, the insufficient sensitivity limits the application of ELISA in field analyses that demand rapid results. Therefore, improvement of sensitivity and reduction in the analytical time of the current ELISA method are highly required.

Recently, nanoparticles have drawn increased attention in developing simple and sensitive immunosensing platforms because of their high surface areas and physicochemical properties^13–16^. Typically, magnetic beads can be used as carriers of antibodies to specifically capture and accumulate targets from complex samples. The targets are easily separated from the reaction mixtures in the presence of a magnet^17^. Gold nanoparticles (AuNPs) have been widely used in chemical and biological assays because of their facile synthesis, high chemical stability, large specific surface area, and biocompatibility^15,18,19^. The AuNP can conjugate many signal molecules, which enables signal amplification. For instance, AuNPs conjugated to horseradish peroxidase (HRP)-labeled antibodies have been applied in immunoassays^20^. This strategy has been proven to significantly improve the detection limit. However, in this strategy, the detector antibody is required to firstly conjugate with HRP through a tedious and high-cost procedure.

In our previous work, dual-functionalized AuNPs have been prepared by simultaneously tagging HRP and antibody on AuNPs via a simple and low-cost procedure and used to construct a portable electrochemical immunosensor for GM crop detection^21^. The results indicated that as-prepared dual-functionalized AuNP nanoprobes can significantly enhance detection sensitivity. However, this immunosensor demands a complicated and costly manufacturing process. Herein, we developed an exceptionally simple and sensitive immunosensing platform targeting Cry 1Ab for the verification of GM crops based on a AuNP-triggered enzyme signal amplification system and immunomagnetic separation strategy. In this investigation, both anti-Cry 1Ab monoclonal antibody and HRP were independently combined onto AuNPs. As-prepared dual-functionalized AuNPs were employed as signal amplification probes to enhance detection sensitivity. Magnetic beads were used as the carriers of anti-Cry 1Ab polyclonal antibodies to prepare the capture probes. In the presence of Cry 1Ab, the capture probe and signal probe simultaneously immunoreacted with the protein to form a sandwich immunocomplex, following the removal of unbound components by a magnetic field, the one-step detection of GM crops was achieved. Chemiluminescent (CL) detection is believed to be one of the most sensitive technology because it occurs by the oxidation of a luminescent substrate, such as luminol, without an excitation source, thus results in a lower background and an enhanced sensitivity. Owing to the application of HRP as the signal probe, CL as well as colorimetric methods could be adopted to quantitatively detect GM crops with a high sensitivity.

## Materials and Methods

### Materials and apparatus

PM3-050 magnetic beads modified with carboxyl groups (750 nm, 10 mg mL^−1^) were purchased from Shanghai Allrun Co., Ltd. (China). HAuCl_4_·3H_2_O, and trisodium citrate were all supplied by Sinopharm Chemical Reagent Co., Ltd. (China). *N*-hydroxysuccinimide (NHS), 1-ethyl-3-(3-dimethylaminopropyl) carbodiimide (EDC), streptavidin-HRP, and 2-(*N*-morpholino) ethanesulfonic acid monohydrate (MES) were all purchased from Sigma-Aldrich Chemical Co., Ltd. (USA). Cry1Ab was provided by Case Western Reserve University. CP4-EPSPS protein, PAT/*bar* protein and PAT/*pat* protein were all provided by YouLong Biotechnology Co., Ltd. (China). Monoclonal and polyclonal antibodies for Cry 1Ab, HRP-labeled monoclonal antibody for Cry 1Ab were all obtained from Riogene Inc. (China). Bovine serum albumin (BSA) was supplied by Biosharp Co., Ltd. (China). An ELISA kit for Cry 1Ab was purchased from Envirologix Inc. (USA). Cry 1Ab was diluted with phosphate buffer (PB) at 0.010 M and pH 7.4 containing 1.0% BSA. 3,3’,5,5′-Tetramethylbenzidine (TMB) substrate for colorimetric detection consisted of solution A and solution B (Shanghai Macklin Biochemical Co., Ltd, China). HRP substrate for CL detection consisted of two solutions. Solution 1 contained 5.0 × 10^−5^ M luminol (Sigma-Aldrich, USA) and 5.0 × 10^−5^ M *p*-iodophenol (Sigma-Aldrich, USA) in 0.10 M PB at pH 8.5. Solution 2 contained 1.0 × 10^−2^ M H_2_O_2_ (Sinopharm Chemical Reagent Co., Ltd., China). Other reagents and solvents were of analytical grade and were purchased from Sinopharm Chemical Reagent Co., Ltd. (China). All solutions were prepared using 18.2 MΩ water obtained by a Millipore-XQ system.

Genuine GM rice and maize samples carrying a *cry 1Ab* gene were collected and identified by the Supervision and Test Center (Wuhan) for Environmental Safety of Genetically Modified Plants of the Chinese Ministry of Agriculture and Rural Affairs. Non-GM crop samples were purchased from the local market.

The absorbance measurements were performed with a Model 680 microplate reader (Bio-Rad, USA). The ultraviolet visible (UV-vis) absorption spectra were obtained from a SPECORD 205 UV-vis spectrophotometer (Analytik Jena, Germany). The measurement of particle size distributions was carried out with a Zetasizer Nano ZSP (Malvern Panalytical, UK). The CL measurements were performed with MPI-A CL analyzer (Xi’an Remax Electronic Science & Technology Co., Ltd., China) equipped with a photomultiplier biased at −800 V.

### Preparation of genuine samples

GM crop seed or leaf sample was first ground into a power. Then 0.020 g of GM crop powder was suspended in 1.0 mL of ultrapure water, followed by shaking for 10 min and stewing for overnight at 4 °C. After centrifugation at 8000 rpm for 5 min to discard the precipitates, the resulting solution was stored at 4 °C for further use.

### Preparation of immunomagnetic beads

Two hundred microliters of 10 mg mL^−1^ of carboxylated-magnetic beads was first washed twice by using 500 µL of MES buffer at 10 mM and pH 6.0 containing 0.050% Tween 20. Then, 400 µL of 5.0 mg mL^−1^ of NHS and 5.0 mg mL^−1^ of EDC solution (v/v = 1:1) was added to the above solution. After activation for 30 min at 37 °C, the excess EDC, NHS and byproducts were removed via magnetic separation using a magnetic scaffold. After thorough washing, 200 µL of polyclonal antibody for Cry 1Ab at 2.0 mg mL^−1^ was added to the activated magnetic beads. The mixture was gently stirred to react for 4 h at 37 °C. Finally, the immunomagnetic beads were separated from the free antibody, resuspended in 1.0 mL of 10 mM PB buffer at pH 7.4 containing 1.0% BSA and 0.050% Tween 20 (PBT), and stored at 4 °C for further use.

### Preparation of dual-functionalized gold nanoparticles

Citrate-stabilized AuNPs were prepared according to a previous study^22^. Specifically, 99 mL of deionized water and 1.0 mL of 1.0% HAuCl_4_ were mixed and boiled, followed by quick addition of 2.0 mL of 1.0% trisodium citrate solution. Heating continued for 10 min after the solution color remained unchanged. The prepared AuNP solution was then cooled to room temperature (RT), and stored at 4 °C for further use.

For preparation of the dual-functionalized AuNP composite, the pH of 1.0 mL of AuNP solution was adjusted to 8.5 by adding 0.20 M K_2_CO_3_ solution, followed by centrifugation at 9000 rpm for 30 min to remove the supernatant. Subsequently, 50 µL of 1.0 mg mL^−1^ streptavidin-HRP, 20 µL of 3.0 mg mL^−1^ monoclonal antibody for Cry 1Ab and 250 µL of PB buffer were pipetted into the centrifuge tube containing the AuNP pellet. The mixture solution was then stirred for 1 h at 25 °C and allowed to stand for 1 h at RT. After that, 30 µL of 5.0% PEG20000 was injected into the resultant mixture, followed by stirring overnight at 4 °C. Finally, AuNP composite was purified and washed with 2.0 mL of 10 mM Tris-HCl buffer at pH 7.8 containing 10% sucrose, 2.0% BSA, 10% PVP, 10% PEG8000 and 0.15% Tween-20 for three times. AuNP composites were collected via centrifugation at 9000 rpm at 4 °C for 30 min. The resulting conjugate was redispersed in 1.0 mL of the washing buffer and stored at 4 °C until use.

### Assay procedure

Typically, 5.0 μL of the immunomagnetic bead solution, 100 μL of dual-functionalized AuNP probes and 395 μL of antigen samples diluted with the PB buffer containing 0.10% BSA were pipetted into a centrifuge tube. The mixture solution was gently shaken for 1 h at 37 °C, followed by placement on a magnetic scaffold. After separation, the immunocomplex was washed 3 times with PBT buffer.

For colorimetric detection, 50 μL of solution A and the same amount of solution B were added into the above immunocomplex and incubated for 15 min in the dark. Finally, the absorbance at 450 nm was recorded after the termination of the reaction with 50 μL of 2.0 M H_2_SO_4_. For CL detection, 80 μL of solution 1 was pipetted into the above immunocomplex and the CL responses were collected at 30 s after injection of 20 μL of solution 2.

## Results and Discussion

### Characterization of dual-functionalized AuNPs

**Figure 1:**
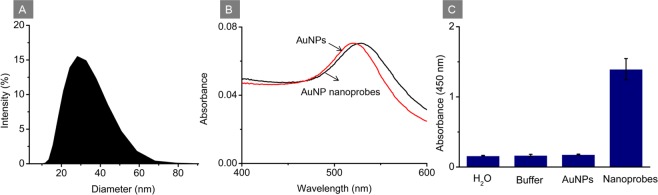
(**A**) Particle size distributions of AuNPs. (**B**) UV-vis absorption spectra of bare AuNPs and AuNP nanoprobes. (**C**) Absorbance response of H_2_O, AuNPs-dissolved buffer, bare AuNPs and AuNP nanoprobes after reaction with TMB solution and H_2_SO_4_ stopping solution

Particle size distribution of AuNPs was investigated and shown in Fig. 1A. According to Fig. 1A, the prepared AuNPs showed an average diameter of 22 nm. UV-vis absorption spectra of AuNPs and AuNP nanoprobes were recorded as shown in Fig. 1B. It was found that the absorption peak of dual-functionalized AuNPs shifted from 520 nm to 529 nm in comparison with that of bare AuNPs. This red shift of the maximum absorption peak demonstrates the successful conjugation of protein at the AuNP surface. To further verify the existence of HRP on the dual-functionalized AuNPs, 0.20 μL of nanoprobes was added into TMB solution followed by the addition of H_2_SO_4_ stopping solution. Afterwards the absorbance of the mixture at 450 nm was recorded and displayed in Fig. 1C. According to Fig. 1C, the absorbance of AuNP nanoprobes was around 1.5 while the absorbance of water, dissolved buffer and bare AuNPs were around 0.20, indicating the existence of HRP conjugated on the AuNP nanoprobes and the resulted pronounce catalytic effect.

### Principle of the proposed immunosensing platform

**Figure 2:**
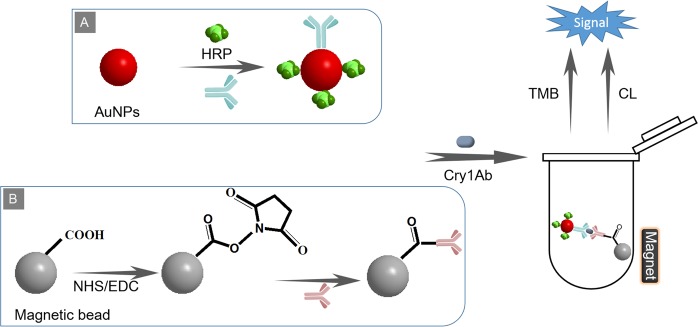
Schematic illustration of a magnetic bead-based immunosensing platform for the one-step detection of Cry 1Ab using dual-functionalized AuNPs.

The principle of the proposed immunosensing platform for the one-step detection of Cry 1Ab is described in Fig. 2. In this approach, the detector monoclonal antibody and HRP were labeled on the surface of the AuNPs by a one-step process. The captured polyclonal antibody was tagged to the magnetic bead particles using the NHS/EDC method. Immunomagnetic beads, Cry 1Ab and dual-functionalized AuNP probes formed a sandwich immunocomplex by specific recognition between the antigen and antibody. By virtue of magnetic separation, the sandwich complex was separated from the complex matrix. The TMB colorimetric signal of the colorful products resulted from the oxidation of TMB catalyzed by HRP molecules in the immunocomplex showed a good linear correlation with the amount of the analyte. In addition, the luminol CL signal was also developed by adopting luminol-H_2_O_2_-*p*-iodophenol system via HRP catalyzation and exhibited a linear correlation with analyte amount. The proposed immunosensing platform targeting Cry 1Ab could be developed for measuring insect-resistant crops.

### Optimization of detection conditions

**Figure 3:**
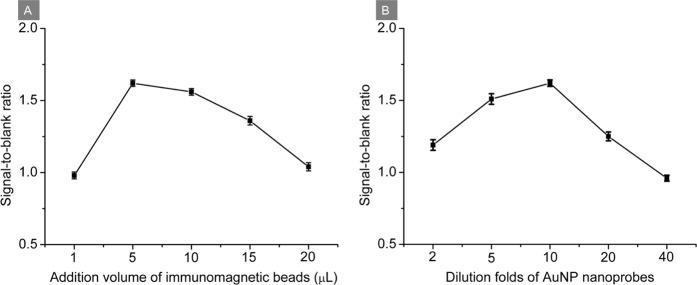
Signal-to-blank ratio (**A**) at the indicated addition volume of immunomagnetic and (**B**) in the presence of AuNP nanoprobes at the indicated dilution folds. Signal refers to the absorbance of 10 ng mL^−1^ Cry 1Ab and blank refers to the absorbance of PB buffer containing 0.10% BSA.

Magnetic beads were employed as magnetic carriers in this immunosensing platform. A small amount of the immunomagnetic beads cannot capture sufficient analytes, resulting in poor analysis sensitivity, while a large amount leads to high nonspecific adsorption, resulting in a false positive. Different addition volumes of immunomagnetic beads (1.0, 5.0, 10, 15 and 20 μL) were investigated to obtain the optimal sensitivity for Cry 1Ab detection. A high signal-to-noise ratio was obtained when the addition volume of immunomagnetic beads was 5.0 μL (Fig. 3A). Thus 5.0 μL of immunomagnetic beads was used for the following study.

The concentration of dual-functionalized AuNP probes was another key factor that affected the sensitivity of the proposed immunoassay. The effect of dilution of the dual-functionalized AuNPs on the absorbance signal was studied utilizing a PB buffer containing 0.10% BSA (blank) and 10 ng mL^−1^ Cry 1Ab (signal). As shown in Fig. 3B, the signal-to-blank ratio reached a maximum value with a dilution ratio of 1 to 10 for the AuNP probes, which was beneficial to the sensitivity of the developed immunosensing platform and thus was adopted for further experiments.

### Analytical performance for Cry 1Ab

**Figure 4:**
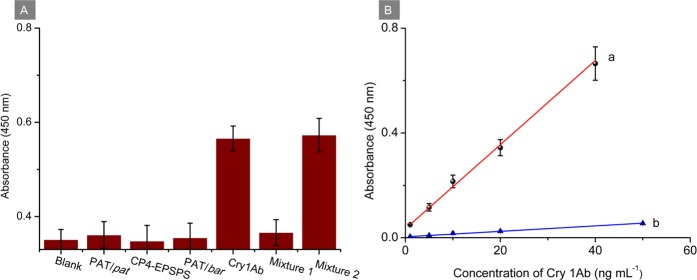
(**A**) Specificity of developed immunosensing platform. (**B**) Dose-response curves for Cry 1Ab assay based on colorimetric detection using (a) AuNP nanoprobes and (b) HRP-labeled antibody. Error bars are standard deviations of five repeated measurements.

According to Fig. 4B (curve a), a dose-response calibration curve for Cry 1Ab assay based on colorimetric detection was observed in the range of 1.0–40 ng mL^−1^. All the absorbances were calibrated by subtracting the blank response. The linear regression equation was expressed as △*I* (a. u.) = 0.0161 *C* (ng mL^−1^)* + *0.0340 (△*I* represents the absorbance signal; *C* represents the concentration of Cry 1Ab), with a correlation coefficient (*R*^2^) of 0.9952 and the limit of detection (LOD) of 0.50 ng mL^−1^ at a signal to noise ratio of 3. In order to confirm the sensitivity of the developed AuNP-nanoprobes-based approach, the commercial HRP-labeled monoclonal antibody was used to replace the AuNP nanoprobes in a same colorimetric assay. As shown in curve b of Fig. 4B, the increase rate of absorbance upon the increase of Cry 1Ab concentration in AuNP-nanoprobes-based approach was 15.3 times higher than that using HPR-labeled antibody, demonstrating the increased sensitivity.

**Figure 5:**
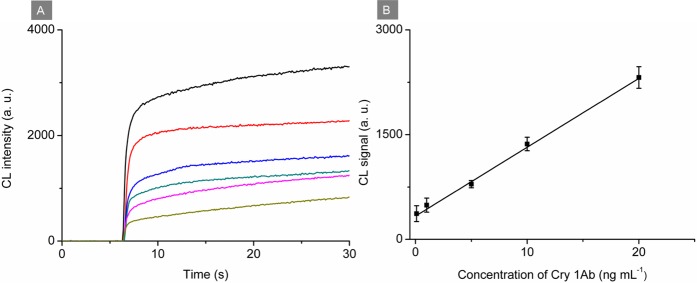
(**A**) CL responses of the developed immunosensing platform for analyzing Cry 1Ab at concentrations of 0, 0.10, 1.0, 5.0, 10 and 20 ng mL^−1^ from bottom to top. (**B**) Calibration curve. Error bars are standard deviations of five repeated measurements

Since HRP was used as the signal tracer in this work, CL method could also be applied for Cry 1Ab detection based on luminol-H_2_O_2_-*p*-iodophenol system with a similar assay procedure. The dose-response calibration curve for Cry 1Ab assay based on CL detection was presented in Fig. 5. All the quantificational data for CL signals were calibrated by subtracting the background. CL signals increased linearly with the increase of Cry 1Ab concentration ranging from 0.10 ng mL^−1^ to 20 ng mL^−1^. The linear regression equation was △*Y* (a. u.) = 98.63 *X* (ng mL^−1^)* + *333.3 (△*Y* and *X* are the CL signal and Cry 1Ab concentration, respectively) with *R*^2^ of 0.9904 and LOD of 0.050 ng mL^−1^ at S/N = 3. Compared with previously reported work using HRP-antibody based method for Cry 1Ab detection, the proposed immunoassay showed an improved LOD (Table 1) resulted from the use of dual-functionalized AuNPs. In many countries, the labeling policy of GM organisms stipulated the labeling threshold value, such as 0.9% in EU, 3% in Korea and 5% in Japan. To confirm whether the sensitivity of the proposed method could reach the labeling threshold, GM rice BT63, as a model, was serially diluted to 5%, 0.9%, 0.5% and 0.1% using non-GM rice to perform the developed colormetric and CL assays. As shown in Fig. S1, compared with the non-GM rice, both the colormetric and CL methods generated significant positive signals with the content of GM crops down to 0.1%, demonstrating the proposed immunosensing platform can meet the requirement of the mandatory labeling of GM products.

**Tablee 1:** Comparison of current available ELISA methods for detection of Cry1Ab.

Method	Analyte	LOD	Reference
ELISA	Cry1Ab	1.5 ng mL^−1^	^23^
DAS-ELISA	Cry1Ab	15 ng mL^−1^	^24^
Commercial ELISA kit	Cry1Ab	0.125 ng mL^−1^	^25^
The proposed colorimetric method	Cry1Ab	0.50 ng mL^−1^	This study
The proposed CL method		0.050 ng mL^−1^	

A conventional sandwich ELISA for GM crops demands at least two reaction steps, two wash steps and a special microplate coated with antibody, resulting in long assay times, multiple operation steps and high cost. The developed immunosensing platform using magnetic beads can achieve one-step assay with one reaction step and one wash step in a centrifuge tube without any treatment, which can significantly simplify the assay procedure and shorten the analytical time.

### Specificity, reproducibility and stability of immunosensing platform

To further evaluate the specificity of the proposed immunosensing platform, interferents of various exogenous proteins that are usually expressed in GM crops were investigated, including PAT/*pat*, CP4-EPSPS and PAT/*bar*. A blank signal was obtained from a dilution PB buffer. As presented in Fig. 4A, the absorbance responses from the interferents at 10 ng mL^−1^ showed slight changes in comparison with the blank signal and were much less than that of Cry 1Ab at the same concentration. The following equation was usually adopted to calculate the degree of interference (DI) value of these interferents:

1 $$\mathrm{DI}=\frac{{\rm{A}}-{\rm{C}}}{{\rm{B}}-{\rm{C}}}\times 100 \% $$

where A, B and C represent the absorbance responses from the interfering proteins, Cry 1Ab and the blank, respectively. The DI values for PAT/*pat*, CP4-EPSPS and PAT/*bar* were 4.7%, 1.4% and 1.9%, respectively. In addition, mixture 1 composed of the three interfering proteins (all at 10 ng mL^−1^) and mixture 2 composed of Cry 1Ab and the three interfering proteins (all at 10 ng mL^−1^) were prepared and examined utilizing the developed approach. The DI value for mixture 1 was calculated to be 7.0%. Compared with the absorbance response of Cry 1Ab, only a negligible change of 1.2% was observed from that of mixture 2. Consequently, the developed approach was not prone to interference by these common GM event proteins.

Additionally, Cry 1Ab at the concentrations of 5.0, 10, 20 ng mL^−1^ were measured using colorimetric and CL detection for 5 times of each concentration (Fig. S2). The relative standard deviations (RSDs) of 5.0, 10, 20 ng mL^−1^ of Cry 1Ab in the colormetric detection were 3.0%, 3.1% and 4.0%, respectively. And RSDs in the CL detection for the above concentration of Cry 1Ab were 2.9%, 4.3% and 4.9%, respectively. Furthermore, the storage stability of immunomagnetic beads and dual-functionalized AuNP probes were also evaluated (Fig. S3). The initial absorbance responses only reduced 13.7% and 5.3% after immunomagnetic beads and AuNP probes had been stored at 4 °C for four months and two weeks, respectively. Thus, the reproducibility and stability of the developed immunosensing platform was acceptable.

### Application in genuine GM crop samples

To validate the reliability of the proposed method for GM crop detection, five GM rice seed samples, a GM maize seed sample and a GM maize leaf sample carrying a *cry 1Ab* gene were analyzed using the developed colorimetric immunoassay. The absorbance response of each GM crop organ sample was corrected by subtracting the corresponding response of non-GM crop organ to reduce the interference of matrix effect. The obtained absorbance signal was then interpolated into the calibration plot (Fig. 4B) to calculate the concentrations of Cry 1Ab in GM crops. As shown in Table 2, the concentrations of Cry 1Ab in these genuine GM crops were measured to be 260.3 ± 25.0, 13.1 ± 2.4, 13.2 ± 4.3, 485.4 ± 28.5, 41.2 ± 6.1, 40.2 ± 4.2 and 387.2 ± 37.4 ng mL^−1^. In addition, the results were validated with the commercial ELISA kit. GM crop samples were diluted appropriately prior to the ELISA assay to ensure that the concentration of Cry 1Ab was within the linear detection range. The *R*^2^ of the results obtained from the two methods was 0.9906, demonstrating that the developed immunosensing platform could be employed for the determination of GM crops. Furthermore, known amounts of Cry 1Ab were spiked into these GM crop samples to conduct the recovery tests. As presented in Table 2, the recoveries were all between 91.0% and 103.4%, and all RSDs were less than 9.7%, demonstrating acceptable accuracy of the developed method.

**Table 2:** Detection of insect-resistant crop samples using the proposed method and the ELISA kit (*n = *3).

Insect-resistant crop samples	GM rice seeds					GM maize seed	GM maize leaf
	BT63	1	2	3	4	5	6
Detected by proposed method (ng mL^−1^)	260.3 ± 25.0	13.1 ± 2.4	13.2 ± 4.3	485.4 ± 28.5	41.2 ± 6.1	40.2 ± 4.2	387.2 ± 37.4
Detected by ELISA kit (ng mL^−1^)	273.0 ± 28.1	14.0 ± 1.3	12.0 ± 1.1	503.4 ± 30.0	46.3 ± 5.2	36.4 ± 3.1	375.0 ± 30.3
Added (ng mL^−1^)	50.0	100.0	50.0	50.0	100.0	100.0	50.0
Found (ng mL^−1^)	307.2	116.5	63.2	532.9	135.4	131.2	436.5
RSD (%)	7.6	8.5	9.7	5.3	4.9	7.4	5.9
Recovery (%)	93.8	103.4	100.0	95.0	94.2	91.0	98.6

## Conclusions

In summary, a simple, rapid and sensitive immunosensing platform has been developed for colorimetric and CL detection of GM crops. With the application of the dual-functionalized AuNPs, the proposed immunosensing platform significantly improved sensitivity which was 15.3 times higher than that using HPR-labeled antibody. In addition, by taking advantage of immunomagnetic separation, the developed immunosensing platform achieved one-step detection of GM crops within 1.5 h. And it possessed outstanding advantages in high sensitivity, exceptional simple operation and rapid detection, thus expanding the applications of ELISA approach for GM organism detection.

## Supplementary information

Electronic supplementary information

## Data Availability

All datasets generated during the current study are available from the corresponding author on reasonable request.

## References

[1] Bonfini L (2012). GMOMETHODS: The European Union Database of Reference Methods for GMO Analysis. J Aoac Int.

[2] Rosa SF (2016). Development and applicability of a ready-to-use PCR system for GMO screening. Food Chem.

[3] Jacchia S (2015). International Ring Trial for the Validation of an Event-Specific Golden Rice 2 Quantitative Real-Time Polymerase Chain Reaction Method. J Agric Food Chem.

[4] Yang Y (2018). Development of Certified Matrix-Based Reference Material as a Calibrator for Genetically Modified Rice G6H1 Analysis. J Agric Food Chem.

[5] Wu, Y. *et al*. Development of a general method for detection and quantification of the P35S promoter based on assessment of existing methods. *Sci Rep*, **4**, 10.1038/srep07358 (2014). 10.1038/srep07358 PMC4258656 25483893

[6] Mannelli, I., Minunni, M., Tombelli, S. & Mascini, M. Quartz crystal microbalance (QCM) affinity biosensor for genetically modified organisms (GMOs) detection. *Biosens Bioelectron*, **18**, 129–140, doi:Pii S0956-5663(02)00166-5, Doi 10.1016/S0956-5663(02)00166-5 (2003). 10.1016/s0956-5663(02)00166-5 12485759

[7] Feriotto G, Gardenghi S, Bianchi N, Gambari R (2003). Quantitation of Bt-176 maize genomic sequences by surface plasmon resonance-based biospecific interaction analysis of multiplex polymerase chain reaction (PCR). J Agric Food Chem.

[8] Mariotti, E., Minunni, M. & Mascini, M. Surface plasmon resonance biosensor for genetically modified organisms detection. *Anal Chim Acta*, **453**, 165–172, doi:Pii S0003-2670(01)01458-1, Doi 10.1016/S0003-2670(01)01458-1 (2002).

[9] Freitas M (2016). Highly Monodisperse Fe_3_O_4_@Au Superparamagnetic Nanoparticles as Reproducible Platform for Genosensing Genetically Modified Organisms. Acs Sens.

[10] Huang L (2015). A novel GMO biosensor for rapid ultrasensitive and simultaneous detection of multiple DNA components in GMO products. Biosens Bioelectron.

[11] Guo LH (2009). Capillary Electrophoresis with Electrochemiluminescent Detection for Highly Sensitive Assay of Genetically Modified Organisms. Anal Chem.

[12] Shan GM, Embrey SK, Schafer BW (2007). A highly specific enzyme-linked immunosorbent assay for the detection of Cry1Ac insecticidal crystal protein in transgenic wideStrike cotton. J Agric Food Chem.

[13] de la Escosura-Muniz A, Parolo C, Merkoci A (2010). Immunosensing using nanoparticles. Mater Today.

[14] Penn SG, He L, Natan MJ (2003). Nanoparticles for bioanalysis. Curr Opin Chem Biol.

[15] Dykman L, Khlebtsov N (2012). Gold nanoparticles in biomedical applications: recent advances and perspectives. Chem Soc Rev.

[16] Ashaduzzaman, M. *et al*. On/off-switchable LSPR nano-immunoassay for troponin-T. *Sci Rep*, **7**, 44027, 10.1038/srep44027, https://www.nature.com/articles/srep44027#supplementary-information (2017). 10.1038/srep44027 PMC5382532 28382946

[17] Yang X-Y, Guo Y-S, Bi S, Zhang S-S (2009). Ultrasensitive enhanced chemiluminescence enzyme immunoassay for the determination of α-fetoprotein amplified by double-codified gold nanoparticles labels. Biosens Bioelectron.

[18] Nie L, Liu F, Ma P, Xiao X (2014). Applications of Gold Nanoparticles in Optical Biosensors. J Biomed Nanotechnol.

[19] Oh, S. Y. *et al*. Development of gold nanoparticle-aptamer-based LSPR sensing chips for the rapid detection of Salmonella typhimurium in pork meat. *Sci Rep*, **7**, 10.1038/s41598-017-10188-2 (2017). 10.1038/s41598-017-10188-2 PMC5579046 28860462

[20] Ambrosi A (2007). Double-codified gold nanolabels for enhanced immunoanalysis. Anal Chem.

[21] Gao HF (2019). A portable electrochemical immunosensor for highly sensitive point-of-care testing of genetically modified crops. Biosens Bioelectron.

[22] Brown KR, Fox AP, Natan MJ (1996). Morphology-dependent electrochemistry of cytochrome c at Au colloid-modified SnO2 electrodes. J Am Chem Soc.

[23] Paul V, Steinke K, Meyer HHD (2008). Development and validation of a sensitive enzyme immunoassay for surveillance of Cry1Ab toxin in bovine blood plasma of cows fed Bt-maize (MON810). Anal Chim Acta.

[24] Dong S (2016). Production and Characterization of Monoclonal Antibody Broadly Recognizing Cry1 Toxins by Use of Designed Polypeptide as Hapten. Anal Chem.

[25] Szekacs A, Lauber E, Takacs E, Darvas B (2010). Detection of Cry1Ab toxin in the leaves of MON 810 transgenic maize. Anal Bioanal Chem.

