# Supplementary material for: Highly sensitive immunosensing platform for one-step detection of genetically modified crops
Source: Sci Rep. 2019 Nov 6;9:16117. doi: 10.1038/s41598-019-52651-2 (PMC6834675; doi:10.1038/s41598-019-52651-2)
Supplement: Supplementary file 1 — Electronic supplementary information [file 41598_2019_52651_MOESM1_ESM.doc]

**Electronic** **supplementary information**

Highly sensitive immunosensing platform for one-step detection of genetically modified crops

**Hongfei Gao, Luke Wen, Wei Hua, Jing Tian*, Yongjun Lin***


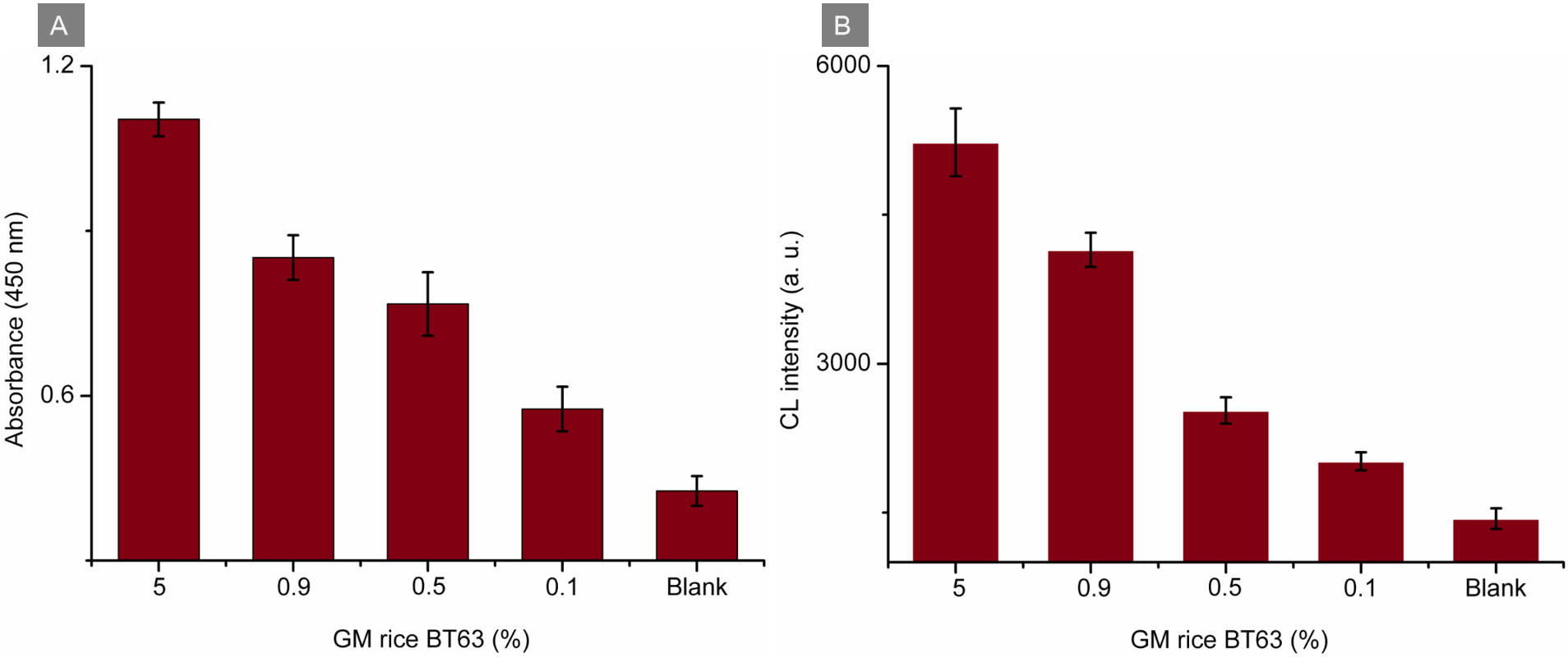


**Figure S1.** Absorbance responses (A) and CL responses (B) of the developed immunosensing platform for GM rice BT63 at the contents of 5%, 0.9%, 0.5%, 0.1% and non-GM rice, respectively. All other conditions were the optimal conditions, *n* = 3.


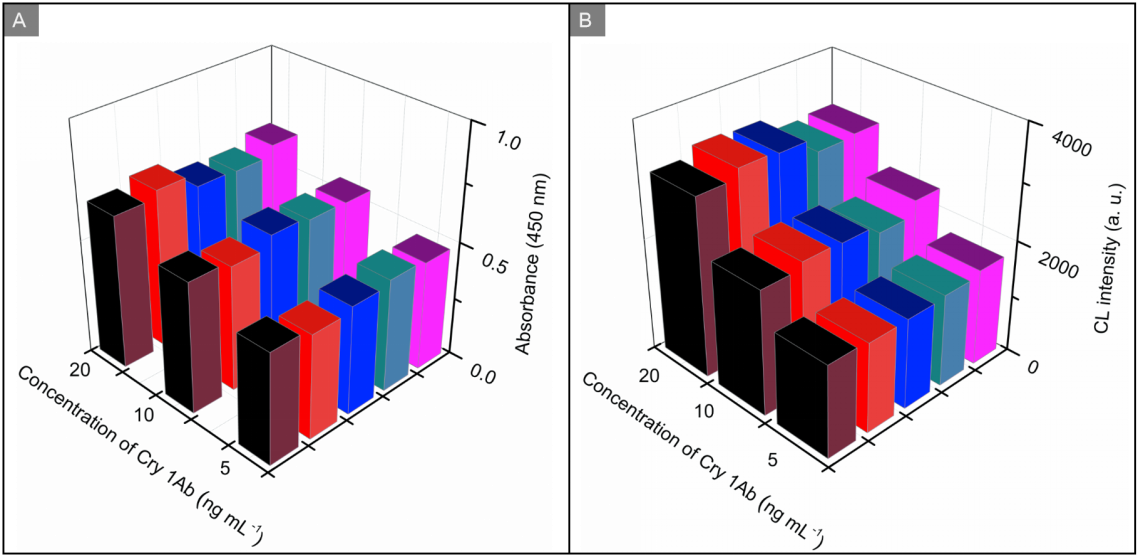


**Figure S2.** Absorbance responses (A) and CL responses (B) of the developed immunosensing platform for Cry 1Ab at the concentration of 5.0, 10 and 20 ng mL^-1^, respectively. All other conditions were the optimal conditions.


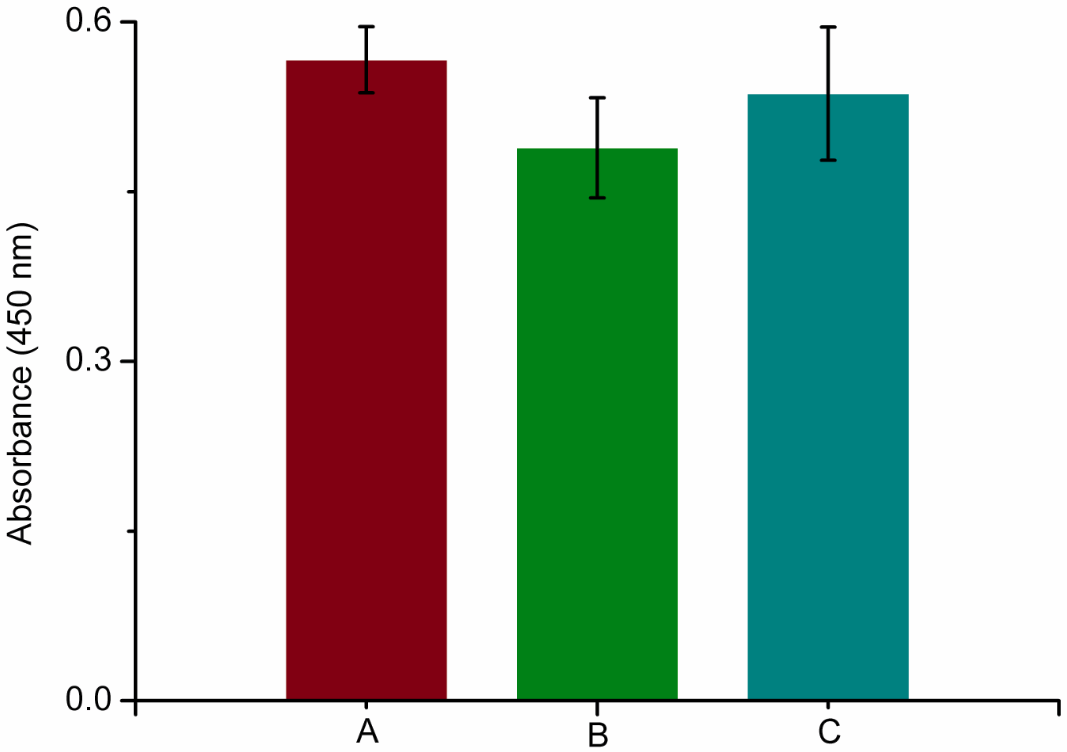


**Figure S3.** The initial absorbance response (A) and the responses after four-month storage of immunomagnetic beads (B) and two-week storage of dual-functionalized AuNP probes (C) of the developed immunosensing platform for Cry1Ab at 10 ng mL^-1^. All other conditions were the optimal conditions, *n* = 3.
